# Is Abusive Supervision the Last Straw? The Buffering Role of Construal Level in the Association of Abusive Supervision With Withdrawal

**DOI:** 10.3389/fpsyg.2022.831185

**Published:** 2022-03-08

**Authors:** Riguang Gao, Bo Liu

**Affiliations:** ^1^School of Business Administration, Jiangxi University of Finance and Economics, Nanchang, China; ^2^School of Foreign Language Studies, Jiangxi Agricultural University, Nanchang, China

**Keywords:** abusive supervision, shame, cognitive-affective processing system, construal level, work withdrawal

## Abstract

Drawing on the theory of cognitive-affective processing system and that of construal level, we propose a moderated mediation model illustrating the relationship among abusive supervision, shame, construal level, and work withdrawal. We tested this model with a two-source time-lagged survey of 387 employees from 129 work teams in central and East China. Results revealed that abusive supervision had a positive association with the emotion of shame and supported the mediating role of shame linking abusive supervision to work withdrawal. Besides, our findings supported the buffering effect of construal level on the shame-work withdrawal relationship as well as the indirect relationship between abusive supervision and work withdrawal channeled through the emotion of shame.

## Introduction

Abusive supervision as a common workplace encounter has received tremendous attention from both academic and practical communities. It was defined by Tepper (2000) as subordinates’ perception of the extent to which supervisors engage in the sustained display of hostile verbal and nonverbal behaviors, excluding physical contact. There is considerable agreement that abusive supervision remarkably correlates with negative outcomes such as turnover intention (Tepper, 2000), emotional exhaustion (Wu and Changya, 2009), and counterproductive work behavior (Mitchell and Ambrose, 2007; Sulea et al., 2013). Work withdrawal, an essential facet of counterproductive work behavior, refers to behaviors that restrict the amount of time working to less than is required by the organization (Spector et al., 2006). If it is not effectively curbed, it will evoke peer’s withdrawal (Eder and Eisenberger, 2007), which will consequently lead to a decrease in productivity (Sagie et al., 2002) and increase in costs to organizations (Carpenter and Berry, 2016; Huang et al., 2020). Though some scholarly efforts have been made to explore the relationship between abusive supervision and work withdrawal (Wei and Si, 2011), what underlies this relationship and what are the boundary conditions remain mysterious (Tepper et al., 2017).

Prior research has examined the relationship between abusive supervision and work withdrawal through emotional mechanisms such as emotion exhaustion and negative emotion in general (Chi and Liang, 2013; Atwater et al., 2015; Huang et al., 2020). However, few studies empirically tested the mediating role of specific negative emotions typically triggered by abusive supervision such as shame (Peng et al., 2019), a type of self-conscious emotions (Kim et al., 2020). We focus specifically on shame rather than other general negative emotions for two reasons. First, shame seems to have a natural connection with work withdrawal, because when subordinates feel ashamed on account of abusive supervision, their desire to hide or disappear will be provoked (Schmader and Lickel, 2006). Second, shame is a very unique and important emotion in organizations. Unlike other emotions, shame can induce a wide range of behavioral responses (such as constructive behaviors on the one hand and withdrawing behaviors on the other) under different circumstances, which can offer significant implications to organizations on how to induce more functional reactions in employees to shame (Daniels and Robinson, 2019). Despite that the presence and consequence of shame at workplace have significant implications for both management practice and scholarly theory, there is a paucity of research examining this specific emotion, not to mention the factors influencing its reactions. Thus, our current study examines the factors influencing the relationship between shame and work withdrawal as a way to echo Daniels’ and Robinson’s (2019) call for more research in this regard.

The theory of cognitive-affective processing system (hereinafter called CAPS) proposes that individuals process information with two separate but interacting systems, called the cool and hot system. When the hot system functions, individuals tend to respond instantly and automatically out of impulse, while when the cool system works, individuals tend to respond calmly out of reason (Metcalfe and Mischel, 1999). CAPS is helpful in explaining people’s emotional and rational responses. However, it fails to delineate the boundary conditions of people’s discrete responses. We argue that people’s discrete responses depend on their construal level (Trope and Liberman, 2000, 2003), conceptualized as the ways that people encode and retrieve information (Wiesenfeld et al., 2017). Since construal level theory (hereinafter called CLT) delineates how different people apply mental representations of different levels of abstraction to the same target, it helps understand people’s difference in self-control (Fujita et al., 2010) and thus helps depict people’s discrete reactions. As a consequence, we propose that subordinates respond differently to shame, elicited by abusive supervision, because they are governed by different processing systems, affected by their construal level.

**Figure 1:**
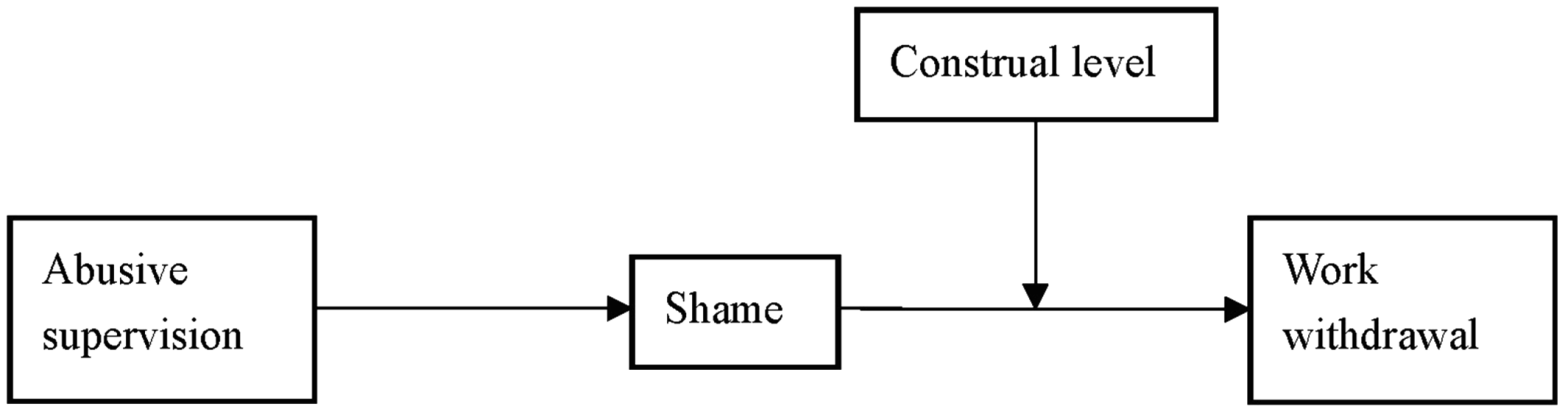
Research model.

Drawing upon CAPS (Metcalfe and Mischel, 1999) and construal level theory (Trope and Liberman, 2000, 2003), we theorize a cross-level moderated mediation model (see Figure 1) to test the relationship between abusive supervision and work withdrawal, mediated by the negative emotion of shame and moderated by construal level. The current study advances the literature in several ways. First, we extend the literature by integrating the theory of cognitive-affective processing system and that of construal level to explore why some subordinates respond more impulsively than others; in another word, why some subordinates withdraw from work to a greater extent than others. This extension is meaningful in that it gives a new perspective on the relationship between abusive supervision and work withdrawal, especially when most research was conducted from the perspectives of emotion-regulation theory, the transactional theory of stress, affective events theory, conservation of resources, etc. (Chi and Liang, 2013; Mawritz et al., 2014; Atwater et al., 2015; Nauman et al., 2020). Second, we expand our understanding of the negative emotion of shame as an underlying mechanism linking abusive supervision and work withdrawal. The extant studies center on either the mediating role of negative emotions in general (Tepper et al., 2017; Yu and Duffy, 2020) or subordinates’ discrete emotions to abusive supervision (Peng et al., 2019), which primarily demonstrated the effect of people’s emotional experiences different in nature and magnitude on different work attitudes and behaviors (Brockner and Higgins, 2001; Zhang et al., 2019), we extend the literature by investigating the unique and understudied emotion of shame (Daniels and Robinson, 2019), and by uncovering why some people respond to shame in an avoidance way (withdrawal), while others do not. Third, by identifying a very important moderator (i.e., construal level), we complement our knowledge on the factors influencing individuals’ discrete behavioral reactions to shame and on the boundary conditions under which the above abusive supervision-work withdrawal relationship is magnified or buffered.

### Emotional Outcome of Abusive Supervision: Shame

The theory of CAPS suggests that there are two separate but interacting hot and cool processing systems within individuals, with the former based on affect and the latter on cognition (Metcalfe and Mischel, 1999). The hot system is to large extent under the control of “stimulus,” driving individuals to respond more rapidly and more automatically (Kross and Mischel, 2010), whereas the cool system is characterized by cognitive rumination, allowing individuals to process the “stimulus” information more slowly and more rationally (Metcalfe and Mischel, 1999). Accordingly, the activation of the hot system contributes to the interpretation of individuals’ emotional reactions related to stimuli. The activation of the cool system, on the other hand, interprets their non-impulsive actions (Metcalfe and Mischel, 1999).

Daniels and Robinson (2019) defined shame in the organizational context as a painful emotion arising when an employee evaluates a threat to the self-concept when he or she has fallen short of an important standard tied to a work-related identity. This definition implies that shame results from a threat to his or her work-related identity, which is tied to feedback from other people, especially from in-group ones (such as leaders) who are in a position to convey more self-relevant information. As Daniels and Robinson (2019) put, the appraisal of deviation from identity-relevant standard and the attribution of deviation to faulty self are the two triggers of shame. Along this line, when subordinates suffer from abusive supervision, their hot system immediately processes the information about this stimulus so that they realize immediately that they fall short of their leaders’ standards and the fact that they are the ones (not someone else) who are abused makes them easily attribute the fault to themselves (Peng et al., 2019), which rapidly leads to their negative emotion, shame.

In short, when subordinates encounter abusive supervision, their hot system will be activated spontaneously because of the self-apparent information exposed by the stimulus, leading to their emotional response of shame because of the perceived threat to self-concept (Daniels and Robinson, 2019; Peng et al., 2019; Kim et al., 2020). In light of this, our first hypothesis proposes a positive relationship between abusive supervision and shame.

*Hypothesis* 1: Abusive supervision is positively related to shame.

### Shame as a Mediator

The extant literature has indicated that negative emotion evokes withdrawal behaviors (Chi and Liang, 2013; Atwater et al., 2015; Liu et al., 2019). Shame, as a particular negative emotion, is held to provoke a desire to hide or disappear (Schmader and Lickel, 2006) in order to avoid a continued threat to the self (Daniels and Robinson, 2019) and thus initiates avoidant responses, such as withdrawal (Nathanson, 1992; Claesson et al., 2007), which encompasses removing from the shame-inducing situation, or minimizing contact with the situation (Chao et al., 2011; Daniels and Robinson, 2019). Combining the strong eliciting effect of abusive supervision on subordinates’ shame with the close association between shame and its common avoidant coping strategy, withdrawal (Daniels and Robinson, 2019), we propose the following hypothesis.

*Hypothesis* 2: Abusive supervision has a positive and indirect relationship with work withdrawal, mediated by Shame.

### Construal Level as a Moderator

One basic premise of CLT is that one’s psychological distance is closely associated with one’s construal level. Specifically, as psychological distance (such as temporal distance, spatial distance, social distance, and hypothetical distance) increases, construal level would become higher, namely, more abstract, and as the construal level increases, the psychological distance people envisage would increase too (Trope and Liberman, 2010). In line with this, the less psychologically distant an event is, the lower levels of abstraction it will be represented (Trope et al., 2007). In another word, when abusive supervision occurs to an individual employee (vs. others), this negative event is taking place right now (vs. distant future), right here (vs. there), implying a close psychological distance. The close psychological distance triggers this individual’s low construal level, drawing his or her attention to the details of what is happening to him or her here and now, which makes him or her unable to transcend the currently experiencing event in time and space (Trope and Liberman, 2010) and thus will instantly activate the hot system, leading this individual to respond reflexively (Metcalfe and Mischel, 1999) and immediately eliciting his or her strong emotion of shame.

Though the construal level can be activated as a state by psychological distance (Trope and Liberman, 2010), it remains relatively stable in people’s mental representation of their work over time (Reyt and Wiesenfeld, 2015). In general, people with higher construal level may attend more to long-term benefits (temporal distance), things happening far away from them (spatial distance), global interests (social distance), and desirability (hypothetical distance) as well, whereas people with lower construal level in contrast focus more on short-term benefits, things happening right in the space, self-interests, and feasibility (Wiesenfeld et al., 2017). As such, we hold that abusive supervision, due to its close psychological distance, leads the abused subordinates to mentally represent the event at a low level of abstraction (i.e., low construal level) and thus to be more susceptible to the control of shame. However, after the activation of the abused subordinates’ hot system, individuals with different construal levels generate different cognitive distractions and thus may exhibit varying degrees of withdrawal behavior (Metcalfe and Mischel, 1999). This is manifested in the following two aspects.

First, construal level affects how subordinates evaluate the rewards, i.e., how valuable and meaningful the desired end-state is. As a result, those with higher construal level attach more cognitive attention to the desirability of the desired end-state (the value of the desired outcomes) rather than its feasibility (Trope et al., 2007; Wiesenfeld et al., 2017). In contrast, those with lower construal level lay more stress on feasibility, namely, how easily a particular end-state can be reached (the difficulty and likelihood of success; Wiesenfeld et al., 2017). Hence, high construal level enables subordinates to be more perseverant and more willing to make efforts to reach their desired end-state (Wiesenfeld et al., 2017), facilitating stronger self-control (Kross and Mischel, 2010), which in turn attenuates the stimulus control. Accordingly, for the abused subordinates with higher construal level, they are more capable of pulling themselves together and more proactive in finding solutions to overcome the negative emotion of shame and to achieve their desired end-state such as self-actualization and career success. They are thus less likely to withdraw. However, those with lower construal level are more sensitive to the feasibility; therefore, the negative emotion of shame haunting them makes them believe that their desired end-state (e.g., career success) is unattainable and feel disenchanted, symbolizing strong stimulus control. In short, the high construal level enables the abused subordinates to stress the desirability of their end-state which empowers them to get rid of the hot system control, beneficial for them to control themselves not to withdraw (Wiesenfeld et al., 2017).

Second, construal level affects how subordinates value their long-term and short-term interests. Specifically, subordinates with higher construal level are more inclined to form abstract representation that transcends the currently experiencing event in time and space (Trope and Liberman, 2010). As a result, subordinates with higher construal level are more likely to concentrate on what is in line with their long-term interests, whereas those with lower construal level are more likely to weigh their short-term concerns (Schmeichel and Vohs, 2009; Wiesenfeld et al., 2017). When subordinates’ long-term interests are in conflict with their short-term concerns such that one behavior is satisfying for the moment but harmful in the long run, higher construal level allows them to attach more attention to the long-term rewards. Close attention to the long-term interests distracts their attention to short-term concerns, which hence leads to greater self-control (Trope et al., 2007; Fujita et al., 2010; Kross and Mischel, 2010) so that subordinates’ cool system is in a position to function. Compared with struggling to advance, withdrawal is much easier and more effortless. In view of this, withdrawal seems to be a comfortable choice in the short term but it will negatively impact subordinates’ job performance (Mobley, 1982) which will ultimately jeopardize their career advancement (Restubog et al., 2011) in the long run. The good thing is that subordinates’ higher construal level allows their cool system to take the place of the hot system to function, which inhibits their impulse of withdrawal by guiding them to put the short-term concern (shame) aside and drawing their attention to the long-term interests (performance improvement, self-actualization, and career success).

Taken together, we hypothesize the moderating effect of construal level on the positive relationship between shame and work withdrawal rather than on the relationship between abusive supervision and shame.

*Hypothesis* 3:  Construal level moderates the relationship between shame and work withdrawal such that subordinates with lower construal level have a stronger tendency to withdraw from work when feeling shame, but those with higher construal level are less inclined to do so.

Based on hypothesis 3 and our previous reasoning on the indirect relationship between abusive supervision and work withdrawal, we further argue that although abusive supervision, as a hot event, can activate hot emotions in subordinates, it does not necessarily cause them to give up their efforts and withdraw from work. The last straw that breaks the subordinates’ back is actually their low construal level. Thus, we theorize the moderated mediation hypothesis as follows.

*Hypothesis* 4:  Construal level moderates the indirect and positive relationship between abusive supervision and withdrawal through shame such that the indirect and positive relationship is weaker for subordinates with higher construal level and that relationship is stronger for subordinates with lower construal level.

## Materials and Methods

### Participants and Procedures

We invited MBA students from three classes to assist our survey. A total of 138 students eventually accepted our invitation. All of them are full-time workers working at small and medium-sized enterprises in central and East China. These students were required to randomly invite one work team at their companies, encompassing one team leader and three subordinates. These MBA students assisted in administering four questionnaires, with one to be filled by the leader and the other three by the subordinates. To safeguard confidentiality, all the questionnaires were put inside envelopes where numbers and alphabets were marked to match the subordinates with their corresponding leaders. At the beginning of each questionnaire, instructions were given to inform the participants of the research purpose and the confidentiality of the survey. They were also informed that they were voluntary and free to express their real feelings, which would be used for research purposes only. Their finished questionnaires were requested to be put in the original envelopes, sealed with their own signatures on, before being handed to the MBA students. Data were collected at two time points, with a 1-month interval. At time 1, subordinates were instructed to rate their leaders’ abusive supervision as well as their own construal level. At time 2, these subordinates were requested to report their emotion of shame. Meanwhile, their corresponding leaders were asked to complete measures of the subordinates’ withdrawal behavior.

We collected valid responses from 414 subordinates at time 1. Among these subordinates, 387 continued on with the survey themselves and were rated by their team leaders at time 2. Consequently, our analysis included a sample of 129 work teams. Among the 129 team leaders, 61.2% were male. Most of them had a bachelor’s degree (62.8%) or master’s degree (26.4%). The vast majority of them were middle managers (62.8%), with the rest being first-line managers (24.8%) and senior managers (12.4%). Among the subordinates, 44.4% were male, 68.7% had a bachelor’s degree, 16.3% had a master’s degree, and 11.9% had a college degree. As for tenure, 68.7% of them had worked for the company for no more than 6 years.

### Measures

Multiple-item scales were utilized to measure the concerned variables, all measures of which (unless otherwise specified) were rated using a 5-point Likert-type scale ranging from 1 (strongly disagree) to 5 (strongly agree). The researchers translated these English scales into Mandarin Chinese, following the back-translation procedure proposed by Brislin (1980).

#### Abusive Supervision (T1)

It was measured using a shortened 5-item scale (Mitchell and Ambrose, 2007) at Time 1 (*α* = 0.94). Sample items are “My supervisor tells me my thoughts and feelings are stupid,” and “My supervisor makes negative comments about me to others.” We applied R_wg(j)_, ICC_(1)_, and ICC_(2)_ to judge the rationality of data aggregation. Statistic results (mean *r*_wg(j)_ = 0.89; median *r*_wg(j)_ = 0.96; ICC_(1)_ = 0.43, ICC_(2)_ = 0.69; *F*_(128, 258)_ = 3.24, *p* < 0.0001) supported our aggregation decision.

#### Construal Level (T1)

To measure subordinates’ construal level, we chose 12 items with the highest loadings from the 25-item scale developed by Vallacher and Wegner (1989). Each item is followed by two alternatives, one representing the “how” aspect of the activity (low-level construal) and the other representing the “why” aspect of the activity (high-level construal). For example, subordinates were required to choose whether “Cleaning the house” was best described as “showing one’s cleanliness” (high construal level) or “vacuuming the floor” (low construal level) and whether “painting a room” was best described as “applying brush strokes” (low construal level) or “making the room look fresh” (high construal level). Alternatives featuring low-level construal was coded as 1, whereas those featuring high-level construal was coded as 2. We averaged the 12-item scores to capture the value of each subordinate’s construal level. The internal consistency (*α*) of this scale was 0.58.

#### Shame (T2)

Following Cavalera et al. (2017) we used a simplified scale of shame to measure the subordinates’ feelings of shame (*α* = 0.95). The short version contained four items, to which we added a time indicator. Sample items are “In the last one month, I felt humiliated, disgraced” and “In the last one month, I felt worthless, powerless.”

#### Withdrawal (T2)

Team leaders rated their subordinates’ behaviors of withdrawal using a simplified version of the subscale of CWB (Atwater et al., 2015). The current version includes three items (e.g., “this employee often comes to work late without permission” and “this employee often stays home from work without legit reason”). Cronbach’s alpha was 0.89 for the withdrawal subscale.

#### Control Variables

In line with the practices of Mawritz et al. (2014), We controlled for subordinates’ age, gender, education, and organizational tenure to rule out the potential influence on our outcome variables, for previous research (Aquino and Douglas, 2003) indicates that these demographic variables offer explanations for different responses to abusive supervision.

## Results

We first conducted CFAs to confirm the construct validity of the concerned variables and to examine the measurement structure by Mplus version 7.4. The baseline model included all the four variables (abusive supervision, shame, withdrawal, and construal level). Results showed that the 4-factor model fit the data well (χ^2^ = 420.94; df = 246; χ^2^/df = 1.71; *p* < 0.001; CFI = 0.96; TLI = 0.96; RMSEA = 0.04; SRMR = 0.05). The hypothesized 4-factor model was superior to other more parsimonious models: a 3-factor model combining shame and withdrawal (χ^2^ = 1962.76; df = 249; χ^2^/df = 7.88; *p* < 0.001; CFI = 0.62; TLI = 0.58; RMSEA = 0.13; SRMR = 0.13), a 2-factor model combining shame, withdrawal, and construal level (χ^2^ = 1285.78; df = 251; χ^2^/df = 5.12; *p* < 0.001; CFI = 0.77; TLI = 0.75; RMSEA = 0.10; SRMR = 0.10), and a 1-factor model with all variables loaded on one factor (χ^2^ = 2748.36; df = 252; χ^2^/df = 10.91; *p* < 0.001; CFI = 0.45; TLI = 0.40; RMSEA = 0.16; SRMR = 0.14). These CFA results suggested that all the 4 key variables had satisfactory discriminant validity. Nevertheless, given the nested structure of our data, we conducted multilevel CFA on the basis of the above conventional CFA. The multilevel CFA results (χ^2^ = 399.25; df = 251; χ^2^/df = 1.70; *p* < 0.001; CFI = 0.96; TLI = 0.95; RMSEA = 0.04; SRMR for within = 0.05; SRMR for between = 0.03) supported the consistency of the factor structure at both levels. Based on these results, we continued on with hypotheses testing using the same software. Table 1 shows the descriptive statistics of the concerned variables and their zero-order correlations.

**Table 1:** Means, standard deviations, reliabilities, and correlations among study variables.

Variables	*M*	SD	1	2	3	4	5	6	7	8
1. Gender	1.56	0.50								
2. Age	2.58	1.14	0.01							
3. Org tenure	2.18	1.30	0.11^*^	0.60^**^						
4. Education	2.98	0.64	0.02	−0.29^**^	−0.21^**^					
5. AS	1.60	0.62	−0.04	−0.03	−0.01	0.05	**(0.94)**			
6. Shame	1.82	0.93	−0.10	−0.04	−0.01	0.05	0.36^**^	**(0.95)**		
7. Withdrawal	1.72	0.98	0.01	−0.06	−0.05	−0.05	0.17^**^	0.23^**^	**(0.89)**	
8. CL	1.42	0.20	0.05	−0.04	0.13	0.02	0.05	0.03	0.05	**(0.58)**

*AS = Abusive supervision, Org tenure = Organizational tenure, and CL = Construal level. N = 387. Cronbach’s alpha values of each scale are boldfaced and noted on the diagonal. Gender: 1 = male, 2 = female; Age: 1 = 25 years and below, 2 = 26 to 30 years, 3 = 31 to 35 years, 4 = 36 to 40 years, 5 = 41 to 45 years, 6 = 46 years and above; Organizational tenure: 1 = 3 years and below, 2 = 4 to 6 years, 3 = 7 to 9 years, 4 = 10 to 12 years, 5 = 13 years, and above*.

* *p < 0.05*;

** *p < 0.01*.

Before testing these hypotheses, we examined the within-group and between-group variances for the mediating and dependent variables (shame and withdrawal) by computing the ICCs. Results (shame: ICC_(1)_ = 0.39, ICC_(2)_ = 0.66; withdrawal: ICC_(1)_ = 0.86, ICC_(2)_ = 0.95) revealed meaningful between-group variances which justified using multilevel analysis (Hofmann, 2002).

We applied the path analysis method in an effort to test our moderated mediation hypotheses (Edwards and Lambert, 2007) in Mplus, where we specified 2 models. Model 1 tests the main and indirect effects of abusive supervision on withdrawal *via* shame. In this model, Hypotheses 1 and 2 are tested. Model 2 tests Hypotheses 3 and 4, concerning the moderating effect of construal level. Following the suggestions of Hofmann and Gavin (1998), we grand-mean centered abusive supervision in all analyses to reduce multicollinearity between random intercepts and slopes.

To test the main and indirect effect of abusive supervision on withdrawal *via* shame, we specified a 2-1-1 model (Preacher et al., 2010), where abusive supervision was specified at the between level, with shame and withdrawal specified at both the between level and the within level. At the between level, abusive supervision was linked to withdrawal directly and indirectly through shame. At the within level, we connected shame with withdrawal. We tested the mediation effect of shame at the between level, because abusive supervision as a constant within a group cannot engender any individual difference within a group (Hofmann, 2002). Consequently, a conventional two-level model with fixed path coefficients (Hofmann, 1997) was specified. In other words, we specified the coefficient of the shame-withdrawal path to be identical at both levels, facilitating the interpretation of the indirect effect.

Table 2 shows the path coefficients for model 1. As it presents, abusive supervision was positively related to shame (*a* = 0.33, *p* < 0.001). Hypothesis 1 was supported. Hypothesis 2 proposes a positive and indirect relationship between abusive supervision and work withdrawal through Shame. To test the statistical significance of the indirect relationship, we first applied the product of the coefficient of the abusive supervision-to-shame path and the coefficient of the shame-to-withdrawal path (MacKinnon et al., 2002) and then used the Monte Carlo simulation method with 20,000 replicates to produce the 95% confidence intervals (hereinafter called “CI”) by R version 1.3.1093. Supporting hypothesis 2, abusive supervision did have a significant indirect relationship with withdrawal *via* shame (ab = 0.02, 95% CI = [0.01, 0.07]; excluding zero; See Table 3).

**Table 2:** Multilevel modeling results.

Variables	Model 1 (Hypothesis 1–2)		Model 2 (Hypothesis 3–4)	
	Shame *coeff* (SE)	Withdrawal *coeff* (SE)	Shame *coeff* (SE)	Withdrawal *coeff* (SE)
Gender	−0.08 (0.08)	0.05 (0.04)	−0.08 (0.08)	0.05 (0.04)
Age	−0.05 (0.05)	−0.03 (0.02)	−0.05 (0.05)	−0.03 (0.02)
Org tenure	0.02 (0.04)	0.01 (0.02)	0.02 (0.04)	0.01 (0.02)
Education	0.00 (0.06)	0.02 (0.04)	0.00 (0.06)	0.03 (0.04)
Abusive supervision	0.33^***^ (0.05)	0.14^+^ (0.08)	0.33^**^ (0.05)	0.14^+^ (0.08)
Shame		0.06^*^ (0.03)		0.06^+^ (0.03)
Construal level (CL)				0.07 (0.10)
Shame^*^CL				−0.06^*^ (0.03)
R^2^	0.01	0.06^*^	0.01	0.08^**^

*N = 387. Unstandardized coefficients and standard errors are presented*.

+ *p < 0.10*.

* *p < 0.05*;

** *p < 0.01*;

*** *p < 0.001*.

**Table 3:** Conditional indirect effects of abusive supervision on withdrawal.

Mediator	Moderator: Construal Level	Indirect Effect^a^	S.E.	95%CI^b^
Model 1				
Shame	N/A	0.02	0.01	[0.01, 0.07]
Model 2				
Shame	High (+1SD)	0.015	0.01	[−0.01, 0.04]
	Low (-1SD)	0.022	0.01	[0.003, 0.04]

a *The product of coefficients approach was used to estimate the indirect effects*.

b *95% confidence intervals were produced based on the bootstrapping approach with 20,000 replicates*.

**Figure 2:**
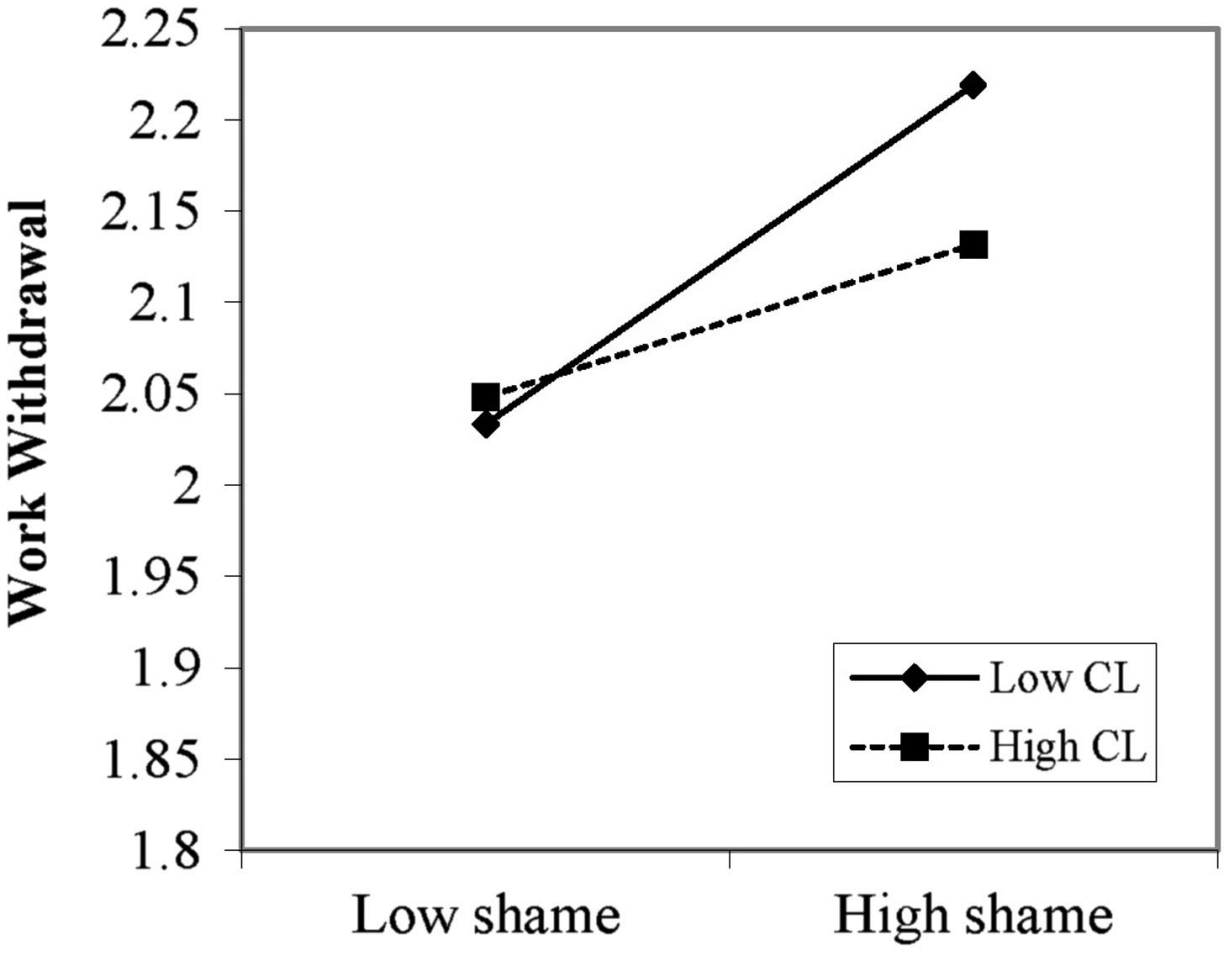
Moderating effect of construal level.

Hypothesis 3 predicts that high construal level attenuates the positive relationship between shame and work withdrawal. Therefore, we expect the interactive effect between shame and construal level on withdrawal to be significant and that the positive relationship to be weaker when construal level is high. Results of model 2 in Table 2 indicate that the direct and positive effect of shame on withdrawal was approaching statistically significant (*b* = 0.06, *p* = 0.05); meanwhile, the interactive effect of shame and construal level was significant in predicting withdrawal (*b* = −0.06, *p* < 0.05). We plotted this interaction in Figure 2. As depicted in Figure 2, low construal level significantly enhances the positive influence of shame on withdrawal (*b* = 0.10, *p* < 0.05), verifying its role of “last straw” that breaks the abused subordinates’ “back,” while if the abused subordinates’ construal level is high, abusive supervision is not likely to break their “back” (*b* = 0.05, n.s.), supporting hypothesis 3.

Hypothesis 4 proposes the moderating role of construal level in the indirect relationship between abusive supervision and work withdrawal through shame. Similarly, we expect this indirect relationship to be stronger among subordinates with lower construal level and weaker among those with higher construal level. As shown in model 2 of Table 3, the indirect effect of abusive supervision on work withdrawal through shame was stronger among subordinates with low construal level (ab_low_ = 0.022, CI[0.00, 0.04]). For those with high construal level, the indirect effect (ab_high_ = 0.015) was not significant with the confidence interval [−0.01, 0.04] containing zero. Besides, the difference in conditional indirect effect was significant at a *p* value of 0.045 (ab_high-low_ = −0.01, 90% CI = [−0.01, −0.00]). Hence, hypothesis 4 received support.

## Discussion

Our study aims to investigate why and when abusive supervision positively correlates with work withdrawal. We drew upon the theory of the cognitive-affective processing system and identified an emotion mechanism of shame underlying this positive relationship. The results of our study indicated that abusive supervision usually energized subordinates’ hot system which aroused their feelings of shame because of the negative implication of self-concept and drove them to respond impulsively, i.e., withdraw from work. But the results suggested that not all subordinates responded the same way. Those with higher construal level responded less impulsively to the emotion of shame, because their construal level empowered them to better control themselves and inhibited the hot system from continuous working. However, the moderating effect of construal level on the indirect relationship between abusive supervision and work withdrawal was only significant among those with low construal level. In spite of the insignificance of its moderating effect among those with high construal level, the differences in conditional indirect effect were significant.

### Theoretical Implications

Our study significantly contributes to the extant literature by providing a new perspective on subordinates’ emotional and behavioral responses to abusive supervision. Prior literature on CAPS stresses the pivotal role of the cool system in cooling the impulse down when individuals are faced with the temptation of immediate gratification (Metcalfe and Mischel, 1999). However, our study expands the literature by empirically proving that the cool system also works in terms of helping individuals overcome obstacles and suppress their need for the immediate release of negative emotion. Additionally, by investigating the moderating role of construal level, our study furthers the current understandings of the boundaries under which these two systems operate. Given the significant moderating effect, construal level has been proven to be one of the determinants of hot-versus-cool-system dominance.

Our research contributes to the literature by uncovering the unique role of the emotion of shame in mediating the effect of abusive supervision on work withdrawal. This contribution is important because most studies ignored the emotional response of shame to abusive supervision. Despite that a limited number of studies (Peng et al., 2019; Kim et al., 2020) have examined this salient emotional reaction, they did not connect feelings of shame with work withdrawal, which was considered to be a shame-focused coping style (Yelsma et al., 2002).

The current study broadens our knowledge about construal level and its moderating role in the relationship between abusive supervision and work withdrawal through the emotion of shame. Wiesenfeld et al. (2017) held that CLT had implications for motivation in terms of impacting what people do and the intensity with which they do it. The application of construal level as a moderator in our study suggested that construal level played a crucial role in explaining why individuals respond differently to threats to their sense of self such as abusive supervision. First, construal level affects whether individuals prioritize desirability or feasibility. Second, it impacts whether individuals put their long-term concerns first or not. Third, it influences whether individuals represent stimulus in a hot way or in a cool way. In a word, CLT sheds light on why some subordinates exhibit more perseverance and resilience than others do when feeling ashamed, elicited by abusive supervision. In this sense, our study answered the recent call for research on the relationship between construal level and perseverance in the service of long-term goals (Wiesenfeld et al., 2017).

### Practical Implications

In addition to the above theoretical implications, our study also yields some practical implications. First, our findings replicate the detrimental effect of abusive supervision on subordinates, which is once again a wake-up call for leaders to be cautious about the harmful impacts of their abusive supervision and to take effective actions to eliminate its occurrence in their management practice. Second, organizations should develop regulations and codes of conduct specifically for leaders to avoid any form of destructive supervision. To what extent leaders’ behaviors are compliant with the codes of conduct should be considered as an appraisal criterion for leaders’ performance. Besides, organizations should warrant a system in place to ensure subordinates’ voices of depression are heard. Otherwise, the subordinates may be worn out by abusive supervision except for those with high construal level. Third, our study proves the buffering impact of construal level, which reminds organizations of measures that can be taken in order to reduce the harmful influence of abusive supervision. Specifically, HR managers should include candidates’ construal level into the selection criteria and design more training programs to elevate employees’ construal level, which is conducive not only for organizations to minimize the potential harm to employees but also for employees to become more perseverant in the pursuit of their long-term goal.

### Limitations and Future Research

As with other studies, our study is subject to some limitations which offer directions for future research.

First, we cannot make a causal inference with a survey design. Nevertheless, we collected data using surveys from different sources (subordinates and leaders) at different time points (two times points with one-month interval). But we still encourage future research to apply multiple methods, including but not limited to experience sampling method (ESM) and experimental design. With ESM, researchers are in a position to observe the daily fluctuations in individuals’ perception of abusive supervision and their levels of shame which aid in detecting subordinates’ transitional trajectories between the hot and cool systems. With experimental designs, researchers are safer to draw causal inferences. Besides, with experimental designs, researchers can apply various methods of manipulations so that they will not be bothered by the relatively low value of Cronbach’s alpha of the construal level in this study, which may be caused by the careless response, reversal ambiguity due to the inclusion of reverse coding items (Weijters and Baumgartner, 2012), and translation nonequivalence due to linguistic and cultural differences (Chang et al., 1999). Therefore, we recommend future research applying more methods of manipulations in the experimental design or doing more pilot studies or analyses of psychometric properties to validate the measure of construal level.

Second, we tested only one negative behavioral outcome of abusive supervision under the theoretical framework of CAPS which delineates the control of two separate systems on individuals’ affect and cognition (Metcalfe and Mischel, 1999). When the cool system is dominant, individuals exhibit rational behavioral responses so that they are liable to persist in the pursuit of long-term goals in the face of obstacles (Metcalfe and Mischel, 1999). Along this line, future research that looks to extend our findings could explore positive behavioral reactions to abusive supervision such as work engagement, creative performance, or job performance in general, demonstrating individuals’ ability to construe events rationally and control themselves from behaving impulsively.

Third, our study focused on only one emotional mechanism that has been proved to have a close association with withdrawal, an avoidance-oriented behavioral reaction. Future research is encouraged to find mediating effects of other avoidance-oriented emotional reactions to abusive supervision (Zhang et al., 2019) such as fear. Besides, future research could give more consideration to individual differences and explore the moderating role of individual differences such as personality traits in the relationship of abusive supervision with emotional responses, for abusive supervision may induce discrete emotions in different individuals. For instance, if the subordinates are high in conscientiousness, when they are abused, they are more likely to feel guilt rather than shame due to the close association between conscientiousness and guilt (Fayard et al., 2012), but they are less likely to withdraw from work due to their features of high responsibility and impulse control (Fayard et al., 2012). But for the subordinates high in Machiavellianism, when they are abused, they may be more likely to feel angry rather than shame and exhibit unethical subsequent behaviors rather than just withdraw from work (Greenbaum et al., 2016).

Finally, we theorized the moderating effect of construal level on the indirect relationship between abusive supervision and work withdrawal through shame. However, our study did not encompass a variety of discrete emotions (e.g., anger and guilt) that are frequently provoked by abusive supervision. Since high construal level plays a critical role in promoting self-control (Fujita et al., 2010). We have every reason to believe that construal level might be equally functional in controlling individuals’ other negative emotional reactions. Hence, we urge future research to explore more possibilities of construal level. Additionally, CLT suggests that four distance cues including temporal, spatial, social, and hypothetical distance initiates high construal level, which in turn distances people from things psychologically (Trope and Liberman, 2010). Future research is also encouraged to examine whether these four distance cues additively or interactively combine to influence individuals’ emotional and behavioral reactions to abusive supervision.

## Conclusion

Negative consequence of abusive supervision is not a new research topic. But it remains unclear how the abused subordinates respond cognitively and emotionally to abusive supervision, what mechanisms underlie their responsive behaviors, and what boundary conditions of these relationships are. Our study answered these questions by integrating the theory of cognitive-affective processing system and that of construal level. We found that abusive supervision was positively correlated with work withdrawal through the emotion of shame and that this indirect relationship was only significant for those subordinates with low construal level, indicating that subordinates’ low construal level might be the “last straw” that breaks the abused subordinates’ “back.” Our study is not an attempt to justify abusive supervision, but rather we hope that our research will arouse more scholarly attention to the buffering effects of subordinates’ construal level on the negative outcomes of abusive supervision.

## Data Availability Statement

The raw data supporting the conclusions of this article will be made available by the authors, without undue reservation.

## Author Contributions

RG and BL contribute to the content of the article and agree to be accountable for it. All authors contributed to the article and approved the submitted version.

## Funding

This work was supported by the National Natural Science Foundation of China (Award number(s): 72162023, 71962015, and 71762016) and Key Base Research Foundation of Humanities and Social Sciences in Jiangxi Universities (Award number(s):JD20031).

## Conflict of Interest

The authors declare that the research was conducted in the absence of any commercial or financial relationships that could be construed as a potential conflict of interest.

## Publisher’s Note

All claims expressed in this article are solely those of the authors and do not necessarily represent those of their affiliated organizations, or those of the publisher, the editors and the reviewers. Any product that may be evaluated in this article, or claim that may be made by its manufacturer, is not guaranteed or endorsed by the publisher.
